# Synthesis and Characterization of Charge-Stabilized Poly(4-hydroxybutyl acrylate) Latex by RAFT Aqueous Dispersion Polymerization: A New Precursor for Reverse Sequence Polymerization-Induced Self-Assembly

**DOI:** 10.1021/acs.macromol.3c00534

**Published:** 2023-06-02

**Authors:** Hubert Buksa, Thomas J. Neal, Spyridon Varlas, Saul J. Hunter, Osama M. Musa, Steven P. Armes

**Affiliations:** † Department of Chemistry, University of Sheffield, Brook Hill, Sheffield, South Yorkshire S3 7HF, U.K.; ‡ Ashland Specialty Ingredients, 1005 US 202/206, Bridgewater, New Jersey 08807, United States

## Abstract

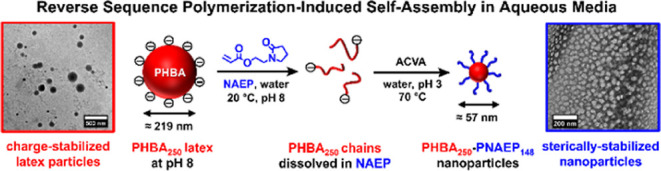

The reversible addition–fragmentation chain transfer (RAFT) aqueous dispersion polymerization of 4-hydroxybutyl acrylate (HBA) is conducted using a water-soluble RAFT agent bearing a carboxylic acid group. This confers charge stabilization when such syntheses are conducted at pH 8, which leads to the formation of polydisperse anionic PHBA latex particles of approximately 200 nm diameter. The weakly hydrophobic nature of the PHBA chains confers stimulus-responsive behavior on such latexes, which are characterized by transmission electron microscopy, dynamic light scattering, aqueous electrophoresis, and ^1^H NMR spectroscopy. Addition of a suitable water-miscible hydrophilic monomer such as 2-(*N*-(acryloyloxy)ethyl pyrrolidone) (NAEP) leads to in situ molecular dissolution of the PHBA latex, with subsequent RAFT polymerization leading to the formation of sterically stabilized PHBA–PNAEP diblock copolymer nanoparticles of approximately 57 nm diameter. Such formulations constitute a new approach to reverse sequence polymerization-induced self-assembly, whereby the hydrophobic block is prepared first in aqueous media.

## Introduction

It is well known that aqueous emulsion polymerization of water-immiscible vinyl monomers offers a versatile route to latex particles.^1^ Indeed, such formulations are used to manufacture vinyl polymers on a scale of millions of tons per annum.^2,3^ Dispersion polymerization has been recognized as a useful alternative to emulsion polymerization since the 1960s.^4,5^ Moreover, dispersion polymerization is applicable to a wide range of solvents, including *n*-alkanes,^6^ alcohols,^7−13^ supercritical fluids,^14−18^ and water.^19−22^ The essential criterion for dispersion polymerization is that the monomer should be miscible with the initial reaction mixture, whereas its corresponding homopolymer should be insoluble. Normally, this scenario would be expected to result in precipitation, but this can be prevented by the inclusion of a suitable polymeric stabilizer to confer steric stabilization, which leads to the formation of microscopic latex particles.^23^ Alternatively, ultrafine nanoparticles can be employed to ensure colloidal stability in some cases.^24^

Aqueous dispersion polymerization formulations based on free radical polymerization are quite rare in the literature. This is mainly because relatively few vinyl monomers are water miscible yet produce a water-insoluble homopolymer. One notable exception is 2-hydroxypropyl methacrylate (HPMA). In 2007, we reported the aqueous dispersion polymerization of HPMA using an azo initiator and employing poly(*N*-vinylpyrrolidone) as a steric stabilizer.^25^ Since then, many aqueous dispersion polymerization formulations based on HPMA (or alternative water-miscible monomers, such as NIPAM, *N*,*N*′-diethyl acrylamide, diacetone acrylamide, or 2-methoxyethyl methacrylate) have been developed by various research groups^26−43^ using reversible addition–fragmentation chain-transfer (RAFT) polymerization, which is a type of pseudo-living radical polymerization.^44−46^ In this case, a suitable water-soluble polymer is first prepared via RAFT solution polymerization: this precursor is then chain-extended via RAFT aqueous dispersion polymerization and acts as a steric stabilizer to prevent macroscopic precipitation. In the case of PHPMA, the weakly hydrophobic nature of this block confers thermoresponsive behavior on the resulting diblock copolymer nano-objects, which can undergo various morphological transitions on adjusting the aqueous solution temperature.^47,48^ Recently, we demonstrated that replacing HPMA with its structural isomer, 4-hydroxybutyl acrylate (HBA), leads to a similar behavior.^49−51^

**Scheme 1:**
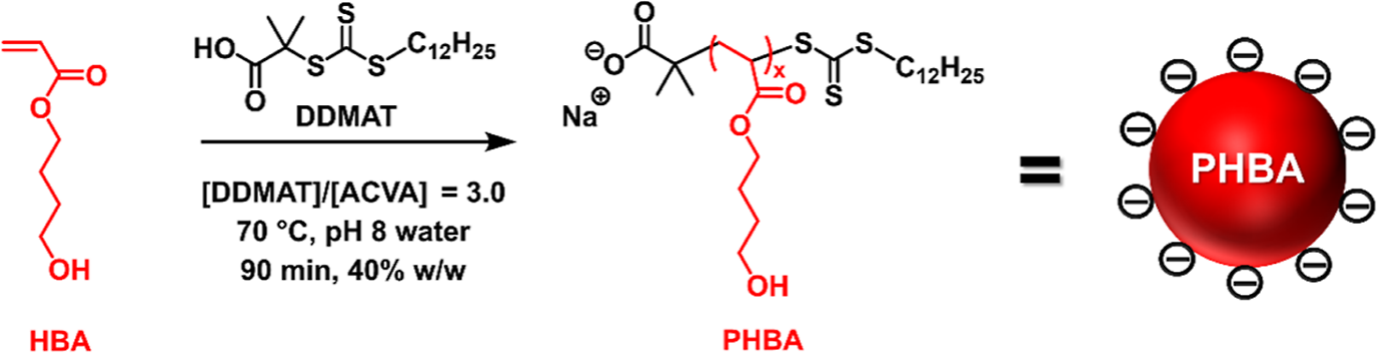
Schematic Representation of the Synthesis of Anionic PHBA Latex Particles via RAFT Aqueous Dispersion Polymerization of HBA at 70 °C Using a Carboxylic Acid-Functionalized RAFT Agent (DDMAT) at 40% w/w Solids and pH 8

Herein we report a new formulation for the RAFT aqueous dispersion polymerization of HBA. Unusually, this involves using a suitable anionic RAFT agent to produce charge-stabilized latex particles (see Scheme 1). Moreover, the weakly hydrophobic nature of the PHBA chains confers stimulus-responsive behavior on such latex particles, which are characterized by transmission electron microscopy (TEM), dynamic light scattering (DLS), aqueous electrophoresis, and ^1^H NMR spectroscopy. Recently, we reported the development of so-called reverse sequence aqueous polymerization-induced self-assembly (PISA) formulations whereby the hydrophobic block is prepared first in the form of PHPMA latex particles, which become the locus for the subsequent polymerization of a suitable water-miscible monomer.^52,53^ Such formulations offer new opportunities for PISA syntheses, not least because the organosulfur-based RAFT groups are located at the end of the steric stabilizer chains rather than within the nanoparticle cores (N.B. Alternative chemistries that can be used for PISA syntheses include atom-transfer radical polymerization,^54^ ring-opening polymerization,^55^ or ring-opening metathesis polymerization).^56^ We briefly demonstrate that the new PHBA latexes prepared in the present study offer new opportunities in this context because they can be molecularly dissolved on addition of a suitable water-miscible monomer prior to reverse sequence PISA. In our initial study, we used a morpholine-based RAFT agent to polymerize HPMA to afford a cationic charge-stabilized PHPMA latex.^52^ Subsequently, this PHPMA precursor was chain-extended using methoxy-capped oligo(ethylene glycol) methacrylate (OEGMA). This monomer diffuses into the latex particles, which are the sole locus for the second-stage polymerization. In contrast, the present study focuses on the polymerization of HBA using a carboxylic acid-functionalized RAFT agent (DDMAT) to afford an anionic PHBA latex. This latex precursor exhibits stimulus-responsive behavior in aqueous solution. In this case, reverse sequence PISA is performed using 2-(*N*-acryloyloxy)ethyl pyrrolidone (NAEP), which leads to molecular dissolution of the PHBA latex particles. Thus, the locus of polymerization is in the aqueous NAEP solution rather than within monomer-swollen latex particles.

## Experimental Section

### Materials

HBA (97% purity) was supplied by BASF (Ludwigshafen, Germany) and purified by exhaustive solvent extraction using *n*-hexane (25 times) to remove diacrylate impurities. CD_3_OD was purchased from Goss Scientific Instruments (Cheshire, UK). Dimethylformamide (DMF) and dimethyl sulfoxide (DMSO) were purchased from Fisher Scientific (Loughborough, UK) and were used as received. The DDMAT RAFT agent, ACVA initiator, glutaraldehyde (50% aqueous solution), D_2_O, and MgSO_4_ were purchased from Sigma-Aldrich (UK) and were used as received. Deionized water was obtained from an Elga Medica DV25 water purification setup and used for all experiments. 2-(*N*-Acryloyloxy)ethyl pyrrolidone (NAEP; 95% purity) was kindly donated by Ashland Specialty Ingredients (Cherry Hill, NJ, US) and purified by dilution with chloroform and washing in 5% aqueous Na_2_CO_3_, saturated aqueous NaCl, and deionized water.

### Methods

#### ^1^H NMR Spectroscopy

^1^H NMR spectra were recorded in CD_3_OD using a 400 MHz Bruker AV3-HD spectrometer with 16 scans being averaged per spectrum. Variable temperature studies were conducted in D_2_O using the same instrument and conditions. All chemical shifts are expressed in ppm (δ).

#### Dynamic Light Scattering

Colloidal dispersions were analyzed at 0.1% w/w solids using a Malvern Zetasizer Nano ZS instrument equipped with a 4 mW He–Ne laser (λ = 633 nm). Scattered laser light was detected at 173°. Either 0.1 M HCl or 0.1 M NaOH solution was used to adjust the dispersion pH. The hydrodynamic *z*-average diameter was calculated using the Stokes–Einstein equation, which assumes perfectly monodisperse, non-interacting spheres.

#### Aqueous Electrophoresis

Aqueous electrophoresis was performed on 0.1% w/w aqueous latex particles (or diblock copolymer nanoparticles) with 1 mM KCl as a background electrolyte using the same Malvern Zetasizer Nano ZS instrument. The initial aqueous dispersion was at pH 6, and this parameter was adjusted using either 0.1 M HCl or 0.1 M NaOH. Zeta potentials (averaged over three consecutive runs) were calculated via the Henry equation using the Smoluchowski approximation.

#### Transmission Electron Microscopy

Cu/Pd TEM grids (Agar Scientific, UK) were coated in-house with a thin carbon film. A single 7 μL droplet of a 0.1% w/w aqueous dispersion of a glutaraldehyde-crosslinked PHBA latex (or glutaraldehyde-crosslinked PHBA–PNAEP diblock copolymer nanoparticles) was pipetted onto the carbon-coated grid and carefully blotted with a filter paper after 1 min. Then, a single 7 μL droplet of a 0.75% w/w aqueous solution of uranyl formate was pipetted onto the grid for 1 min to stain the deposited particles. Excess stain was removed using a vacuum hose. A Philips CM100 transmission electron microscope equipped with a Gatan 1k CCD camera was used to image the stained samples at an accelerating voltage of 100 kV and a beam current of 3 mA.

#### Gel Permeation Chromatography

Molecular weight distributions were analyzed using an Agilent 1260 Infinity gel permeation chromatography (GPC) instrument comprising a 5 μm guard column and two 5 μm Mixed C columns (Polymer Laboratories) connected to a refractive index detector and a UV detector (λ = 305 nm). The eluent was HPLC-grade DMF containing 10 mM LiBr, DMSO was used as a flow rate marker, and the flow rate was 1.0 mL min^–1^. Calibration was achieved using ten near-monodisperse poly(methyl methacrylate) (PMMA) standards ranging from 2,380 to 2,200,000 g mol^–1^.

#### Synthesis of Anionic PHBA Latexes via RAFT Aqueous Dispersion Polymerization of HBA Using a Carboxylic Acid-Based RAFT Agent (DDMAT)

A typical synthesis protocol was conducted as follows. The DDMAT RAFT agent (20.2 mg, 56 μmol), HBA monomer (1.20 g, 8.3 mmol, target DP = 150), and ACVA initiator (5.20 mg, 19 μmol, DDMAT/ACVA molar ratio = 3.0) were added to a 10 mL glass vial. Water (1.80 mL) was added to afford a 40% w/w aqueous solution, which was adjusted to pH 8 using 0.1 M NaOH and deoxygenated with a stream of N_2_ gas for 15 min. The glass vial was immersed in an oil bath set to 70 °C to initiate the RAFT aqueous dispersion polymerization of HBA. This polymerization was allowed to proceed for 90 min. A milky-white PHBA_150_ latex dispersion was obtained with an HBA conversion of at least 97%, as judged by ^1^H NMR spectroscopy studies (the integrated monomer vinyl signals at 5.9, 6.2, and 6.4 ppm were compared to the acrylic backbone signals at 1.5–1.9 ppm). The same protocol was also used to prepare a PHBA_250_ latex using 12.1 mg (33 μmol) DDMAT and 3.10 mg (11 μmol) ACVA initiator.

#### Covalent Stabilization Using Glutaraldehyde as a Crosslinker

Glutaraldehyde (GA, provided as a 50% aqueous solution; 5 μL, 27 μmol) was added to a 0.1% aqueous dispersion of PHBA_150_ latex at pH 6 (6.0 g, 41 μmol HBA; target GA/HBA molar ratio = 0.66) in a 15 mL vial, and this reaction mixture was stirred at 20 °C for 24 h prior to TEM analysis. The above protocol was also used to crosslink the PHBA–PNAEP diblock copolymer nanoparticles.

#### Synthesis of PHBA_250_–PNAEP_148_ Nanoparticles via Reverse Sequence PISA

An aqueous dispersion of 40% w/w PHBA_250_ latex was prepared as reported above and cooled to 20 °C. Then, NAEP (0.900 mL, 4.9 mmol, target DP = 148) and ACVA (3.10 mg, 11 μmol; PHBA/ACVA molar ratio = 3.0) were added to 3.0 g of this PHBA_250_ latex in a glass vial. This reaction mixture was adjusted to pH 3 using 0.1 M HCl and then deoxygenated with N_2_ gas. The vial was placed in an oil bath set at 70 °C, and the reaction mixture was magnetically stirred for 18 h. The final product was a 54% w/w aqueous dispersion of PHBA_250_–PNAEP_148_ nanoparticles (NAEP conversion > 99%).

## Results and Discussion

**Figure 1:**
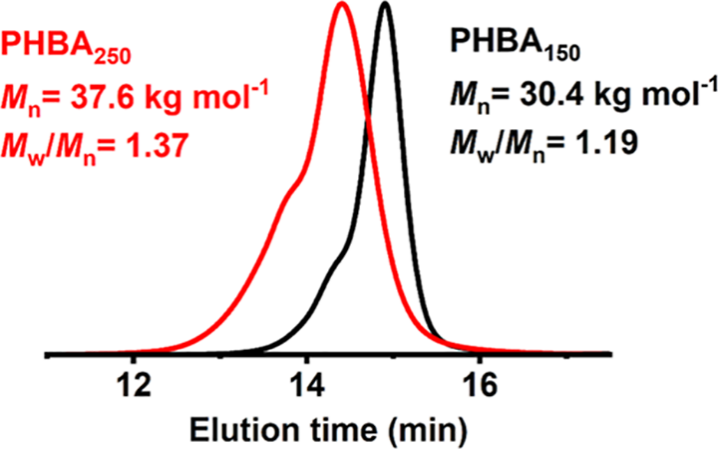
DMF GPC traces recorded using a refractive index detector for two PHBA homopolymers prepared in the form of charge-stabilized latexes via RAFT aqueous dispersion polymerization of HBA at 70 °C using DDMAT as an anionic RAFT agent at pH 8 when targeting 40% w/w solids. The target PHBA DP was either 150 or 250, and a series of PMMA standards were used as for calibration.

Two anionic PHBA_*x*_ latexes were prepared at 40% w/w solids using a carboxylic acid-functionalized RAFT agent (DDMAT) at pH 8 when targeting a PHBA DP of either 150 or 250 (Scheme 1). Although hydrolysis of RAFT end-groups can occur in alkaline aqueous solution, trithiocarbonates appear to be significantly more stable than dithiobenzoates in this context.^57−59^ DMF GPC studies of the PHBA_*x*_ chains using a refractive index detector indicated relatively narrow molecular weight distributions (Figure 1). Targeting a higher degree of polymerization (*x*) resulted in a higher dispersity (*M*_w_/*M*_n_) owing to the appearance of a high molecular weight shoulder. Given that the HBA monomer was extensively purified to remove diacrylate impurities, this feature most likely indicates chain transfer to polymer, which is a well-known phenomenon for acrylic monomers at high temperature.^60^ Nevertheless, reasonably good control can be achieved during the RAFT aqueous dispersion polymerization of HBA under the stated conditions.

**Figure 2:**
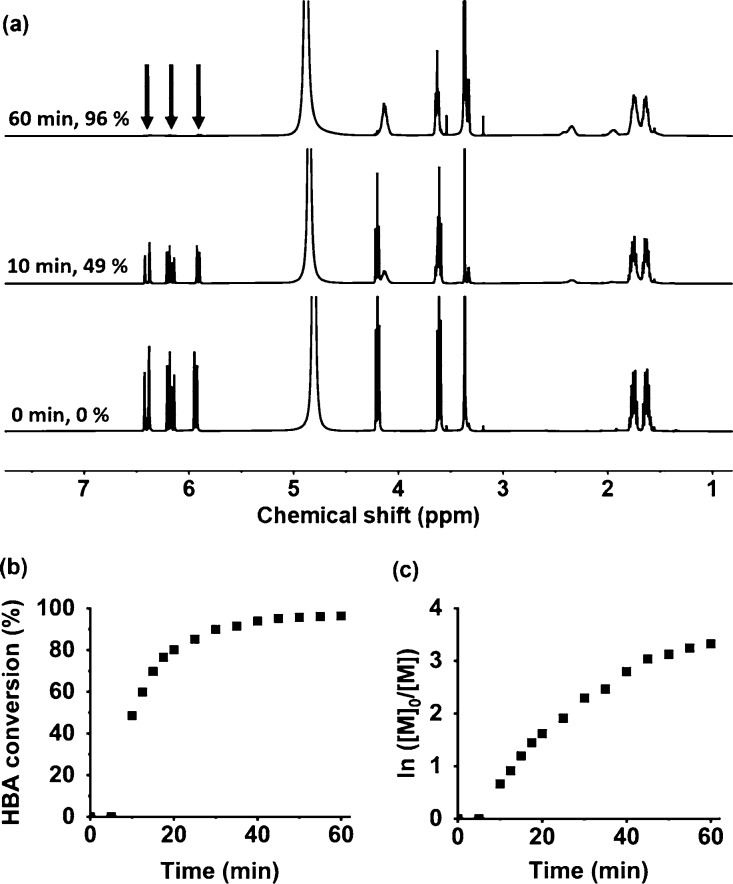
(a) Representative ^1^H NMR spectra recorded at various time points during the synthesis of an anionic PHBA_150_ latex via RAFT aqueous dispersion polymerization of HBA at 70 °C using DDMAT at pH 8 to target 40% w/w solids. Such spectra were used to construct (b) a conversion vs time plot and (c) the corresponding semilogarithmic plot.

^1^H NMR spectroscopy was used to analyze aliquots extracted during the synthesis of a PHBA_150_ latex at pH 8 (Figure 2a,b). An initial 5 min induction period was followed by a relatively fast rate of polymerization, with 90% HBA conversion being achieved within 25 min at 70 °C. Once nucleation occurs, the nascent PHBA particles most likely become swollen with the unreacted HBA monomer, which leads to a relatively high local concentration and hence accounts for the rate acceleration.^32^

**Figure 3:**
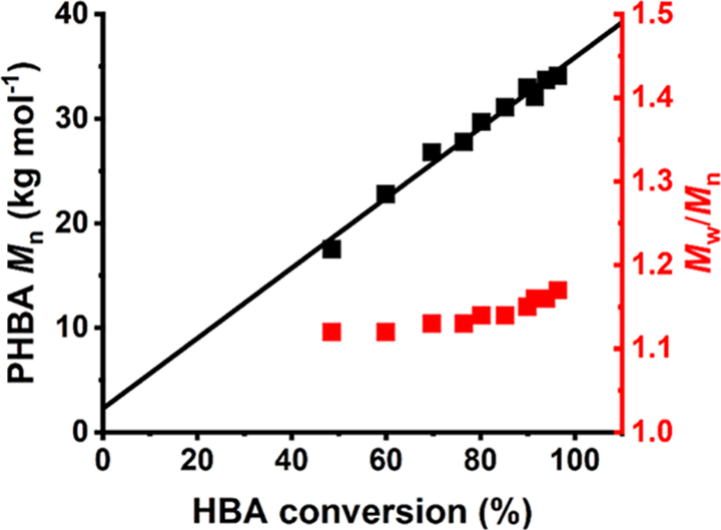
Evolution of *M*_n_ and *M*_w_/*M*_n_ with HBA conversion during the synthesis of a charge-stabilized PHBA_150_ latex at 70 °C using DDMAT at pH 8 when targeting 40% w/w solids.

Nevertheless, DMF GPC analysis of aliquots periodically extracted from the reaction mixture indicated a linear evolution in molecular weight with HBA conversion with relatively low dispersities (*M*_w_/*M*_n_ < 1.20), see Figure 3. These observations confirm that such RAFT aqueous dispersion polymerizations are efficient and exhibit pseudo-living character, as expected for a well-controlled RAFT polymerization.

Aliquots of PHBA_150_ and PHBA_250_ latexes were then diluted from 40 to 0.1% w/w solids using deionized water, and 0.01 M HCl was used to adjust the dispersion pH from pH 8 to pH 6. The resulting dilute dispersions were analyzed by DLS and aqueous electrophoresis. These results, along with ^1^H NMR conversions and DMF GPC data, are summarized in Table 1.

**Table 1:** Summary of NMR, GPC, DLS, and Zeta Potential Data Obtained for PHBA_150_ and PHBA_250_ Latexes Prepared by RAFT Aqueous Dispersion Polymerization of HBA at 70 °C Using DDMAT at pH 8 to Target 40% w/w Solids

	^1^H NMR	DMF GPC		DLS		aqueous electrophoresis
target PHBA DP (*x*)	HBA conversion (%)	*M*_n_ (kg mol^–1^)	*M*_w_/*M*_n_	*D*_h_ (nm)	PDI	zeta potential at pH 6 (mV)
150	97	30.4	1.19	194	0.12	–33
250	98	37.6	1.37	219	0.22	–31

**Figure 4:**
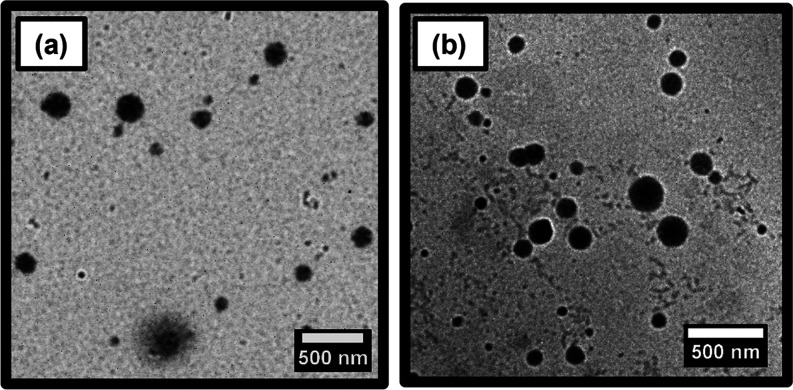
Representative TEM images recorded for (a) PHBA_150_ latex and (b) PHBA_250_ latex prepared at 70 °C after covalent stabilization as 0.1% w/w aqueous dispersions at 20 °C using the glutaraldehyde (GA) crosslinker at a [GA]/[HBA] molar ratio of 0.66.

For TEM studies, each aqueous PHBA latex was diluted to 0.1% w/w solids using deionized water and adjusted to pH 6 using 0.01 M HCl. Covalent stabilization was achieved using glutaraldehyde at a [GA]/[HBA] molar ratio of 0.66 at 20 °C, as reported by Deane and co-workers.^49−51^ Given that PHBA has a relatively low *T*_g_, this crosslinking protocol was essential to prevent film formation during TEM grid preparation. TEM analysis of the resulting crosslinked PHBA_150_ and PHBA_250_ latexes indicated a polydisperse spherical morphology in each case (Figure 4). Using digital image analysis (ImageJ software), the number-average diameters were estimated to be approximately 147 and 155 nm, respectively.

**Figure 5:**
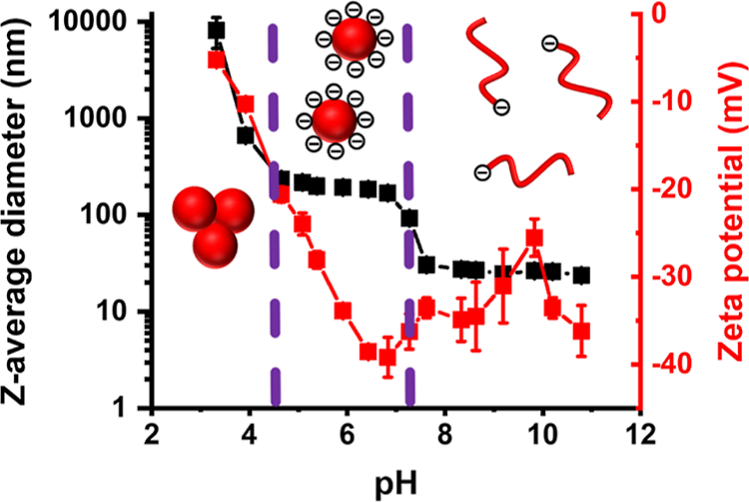
Variation in *z*-average diameter and zeta potential with pH for a 0.1% w/w aqueous dispersion of anionic PHBA_150_ latex particles, as determined by DLS and aqueous electrophoresis, respectively, at 20 °C. Purple dashed lines indicate the onset of latex aggregation (pH < 4.5) and molecular dissolution of the latex particles (pH > 7.2), respectively.

A zeta potential vs pH curve was constructed for a 0.1% w/w aqueous dispersion of the PHBA_150_ latex (Figure 5). As the surface anionic carboxylate groups (p*K*_a_ ∼ 4.5) gradually became protonated, the zeta potential was reduced from around −40 mV at pH 6.5 to approximately −20 mV at pH 4.5 and just −5 mV at pH 3.5. DLS studies indicated aggregation of the PHBA_150_ latex particles on switching from pH 6 to pH 3. On returning to pH 6, most of the latex particles remain aggregated (Figure S1). Thus acid-induced aggregation of the PHBA_150_ latex is irreversible. This is because charge stabilization is no longer effective under such conditions.^61^

Interestingly, raising the dispersion pH above pH 7 reduces the particle diameter from 224 nm to only 19 nm. Moreover, the initially turbid latex dispersion became highly transparent, and the derived count rate was reduced from 440,000 to just 1600 kilocounts per second (kcps), see Figure S2. These observations suggest that latex disassembly occurs in mildly alkaline solution to afford molecularly-dissolved PHBA chains. The pH-responsive behavior of the PHBA_150_ latex is reversible for at least two pH cycles, as indicated by the DLS data shown in Figure S3. More specifically, the molecularly-dissolved PHBA chains formed at pH 8 can be acidified to reform PHBA latex particles at pH 6 (*z*-average diameter = 206 nm). This reformed latex can be redissolved to form PHBA chains again by adjusting the solution pH from pH 6 to pH 8. However, poor reversibility is observed over subsequent pH cycles, possibly owing to the gradual build-up of background salt. This unexpected dissolution presumably reflects the weakly hydrophobic characteristic of the PHBA chains and their relatively short DP of 150.

**Figure 6:**
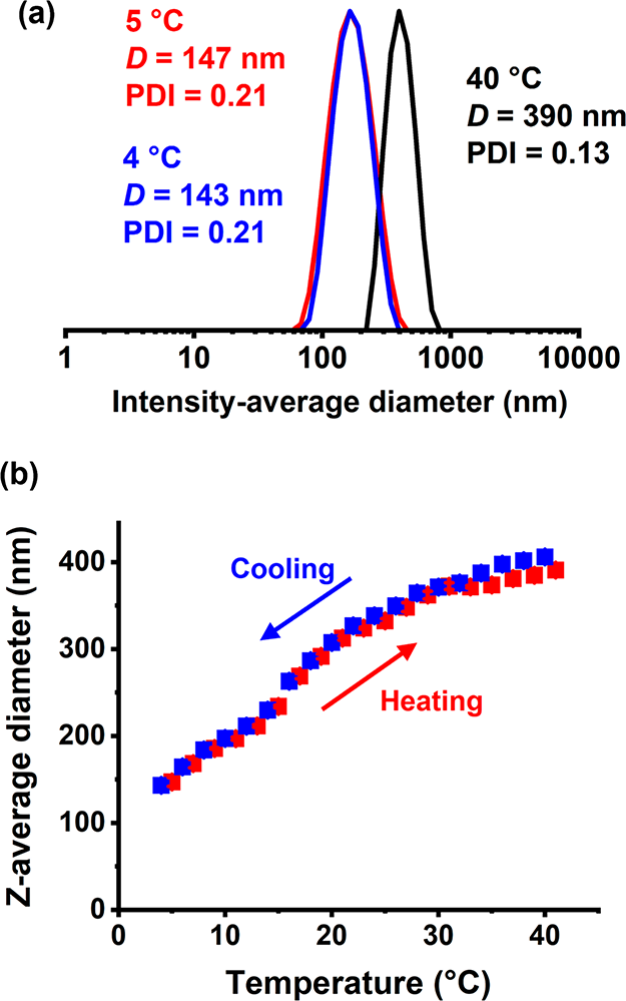
(a) Representative DLS particle size distributions recorded at pH 6 for a 0.1% w/w aqueous dispersion of PHBA_150_ latex (prepared at 40% w/w solids using DDMAT). The red trace was recorded at 5 °C, the black trace after warming to 40 °C, and the blue trace on returning to 4 °C. (b) Variation in *z*-average diameter with temperature obtained for the same PHBA_150_ latex.

A 0.1% w/w aqueous dispersion of the same PHBA_150_ latex was subjected to a thermal cycle at pH 6 (Figure 6a). At 5 °C, its *z*-average diameter was 147 nm (DLS PDI = 0.21). The particle size increased to 390 nm (DLS PDI = 0.13) on warming to 40 °C, while returning to 4 °C produced a *z*-average diameter of 143 nm (DLS PDI = 0.21). The initial and final intensity-average particle size distributions recorded at sub-ambient temperature overlay almost perfectly, suggesting a thermoreversible transition. Subsequently, more detailed DLS studies were conducted (Figure 6b). Again, the *z*-average diameter more than doubled during the heating cycle, from around 150 nm at 5 °C up to almost 400 nm at 40 °C. Such latex swelling corresponds to an approximate 19-fold increase in particle volume. A monotonic reduction in particle diameter was observed during the cooling cycle, with essentially the original particle diameter being regained on returning to 4 °C. These observations are consistent with our recent studies of thermoresponsive PHBA-based diblock copolymer nano-objects in aqueous solutions: ^1^H NMR spectroscopy studies revealed a higher degree of solvation/plasticization of the PHBA chains at elevated temperatures.^50^ The TEM images recorded for the PHBA_150_ latex at pH 6 after glutaraldehyde crosslinking at 5 °C before and after the thermal cycle are shown in Figure S4, providing estimated number-average diameters of approximately 101 and 109 nm, respectively. This is consistent with the thermoreversible behavior indicated by DLS studies (Figure 6). However, a plausible alternative explanation for the change in size indicated by DLS studies could be thermoreversible aggregation of non-swollen PHBA_150_ latex particles.

**Figure 7:**
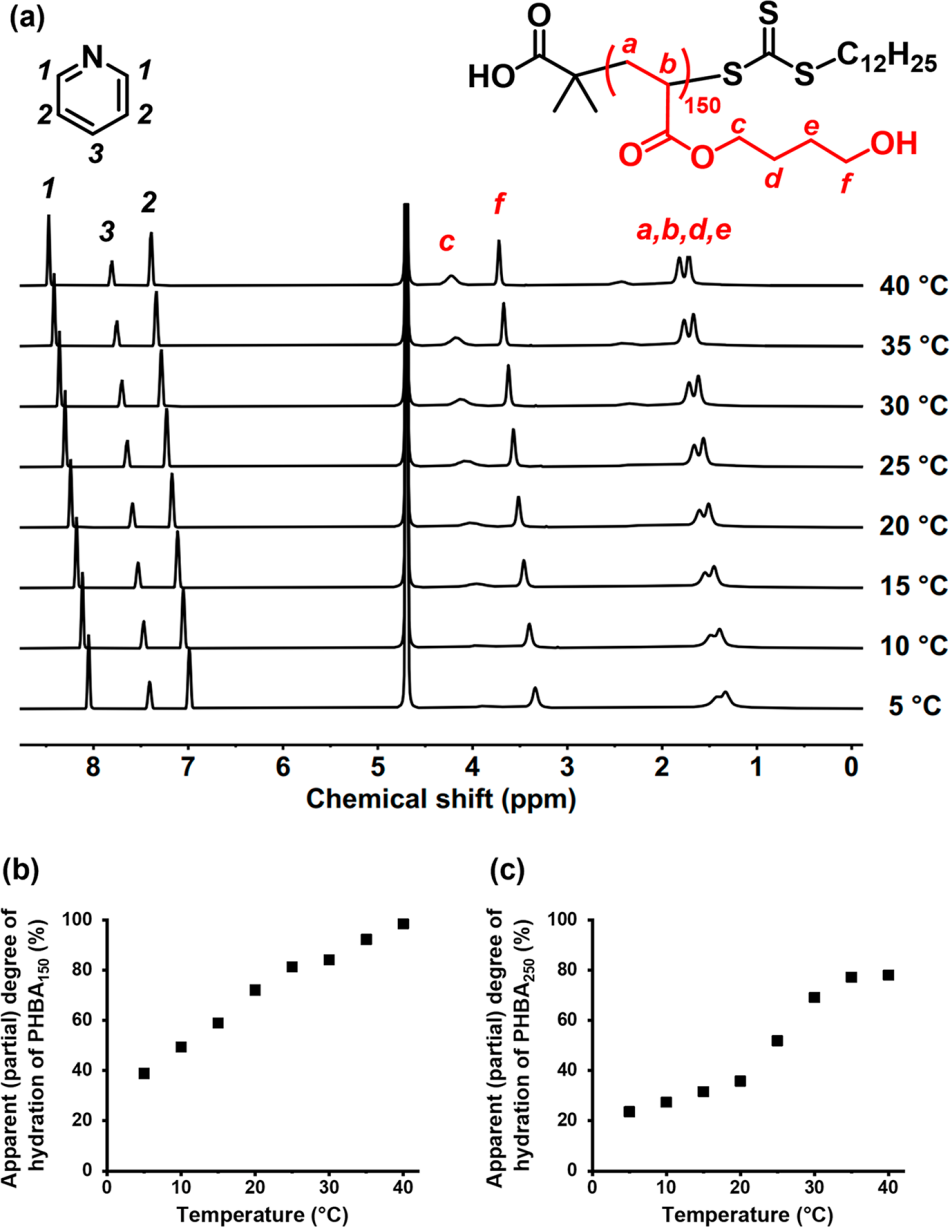
(a) Variable temperature ^1^H NMR spectra recorded on heating a 5.0% w/w aqueous dispersion of the PHBA_150_ latex particles in D_2_O (pH 6) from 5 to 40 °C. (b) All spectra were normalized using pyridine as an external standard, and the apparent (partial) degree of hydration of the PHBA_150_ chains was calculated as a function of temperature. The integrated proton signal *f* was normalized to that observed for the same PHBA_150_ latex after its molecular dissolution in CD_3_OD. (c) Apparent (partial) degree of hydration of the PHBA_250_ chains calculated as a function of temperature. The integrated proton signal *f* was normalized to that observed for the same PHBA_250_ latex after its molecular dissolution in CD_3_OD.

Accordingly, variable temperature ^1^H NMR studies of a 5% w/w aqueous dispersion of PHBA_150_ latex in D_2_O solution at pH 6 were conducted from 5 to 40 °C using pyridine as an external standard (Figure 7a). The pendent methylene proton signals (see *c*, *d*, *e*, and *f* labels) became more intense at higher temperature, indicating a progressive increase in the degree of latex swelling. The apparent degree of hydration of the PHBA chains was estimated by using the pyridine reference signals to normalize the intensity of the *f* proton signal intensity (Figure 7b). The apparent degree of hydration increases from 38% at 5 °C up to 72% at 20 °C. Interestingly, approximately 100% hydration is observed at 40 °C. This temperature-dependent behavior is similar to that reported by Deane and co-workers for PHBA-based diblock copolymer nano-objects.^50^ It is worth emphasizing that PHPMA latex particles do not exhibit any significant change in their degree of hydration despite PHPMA and PHBA being structural isomers.^52^ Thus it is clear that PHBA is even more weakly hydrophobic than PHPMA.

Performing the same variable temperature ^1^H NMR experiments on a 5% w/w aqueous dispersion of charge-stabilized PHBA_250_ latex indicated a lower apparent degree of partial hydration at all temperatures (Figures 7c and S5). For example, the degree of hydration of the PHBA_250_ latex is only 32% at 20 °C, whereas the PHBA_150_ latex is approximately 74% hydrated at this temperature. Hence the thermoresponsive behavior of PHBA clearly depends on its mean DP. In prior studies, we reported that longer PHPMA chains were more hydrophobic (i.e., much less thermoresponsive) than shorter PHPMA chains. Since HBA is a structural isomer of HPMA, it is reasonable to expect that increasing the PHBA DP from 150 to 250 should significantly reduce the thermoresponsive behavior observed for such precursor latexes.^62,63^

**Figure 8:**
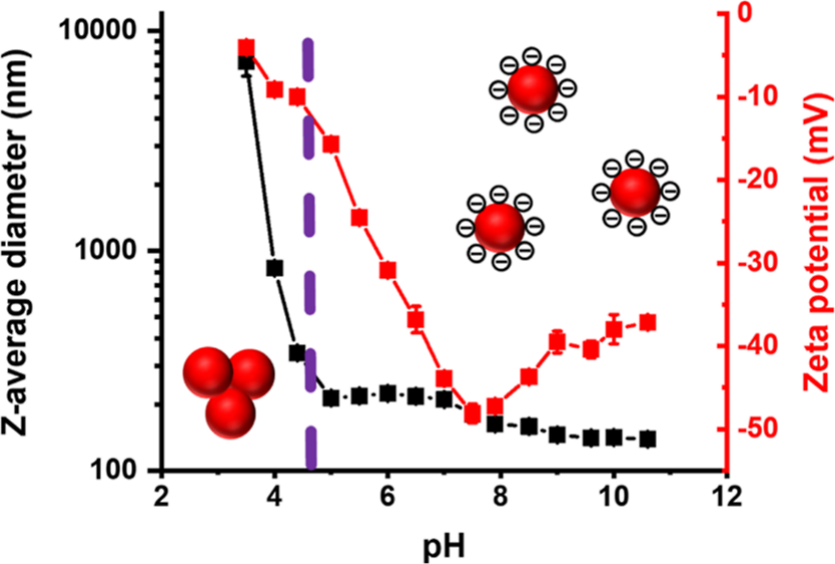
Variation in *z*-average diameter and zeta potential with pH for a 0.1% w/w aqueous dispersion of anionic PHBA_250_ latex particles as determined by DLS and aqueous electrophoresis, respectively, at 20 °C. The dashed purple line indicates the onset of latex aggregation (pH < 4.7).

A zeta potential vs pH curve was constructed for a 0.1% w/w aqueous dispersion of the PHBA_250_ latex (Figure 8). As the surface anionic carboxylate groups gradually become protonated, the latex zeta potential was reduced from −47 mV at pH 7.5 to around −15 mV at pH 4.7 and to just −5 mV at pH 3.5. Again, latex aggregation occurred when the dispersion pH was adjusted below the known p*K*_a_ for isolated carboxylic acid groups, as indicated by the dramatic increase in the apparent *z*-average diameter. Moreover, DLS studies indicated that such aggregation was irreversible (data not shown). However, in contrast to the PHBA_150_ latex (Figure 5), raising the dispersion pH of the PHBA_250_ latex above pH 7 did not cause molecular dissolution. The *z*-average particle diameter was reduced from 220 to 150 nm, but the scattered light intensity remained relatively high. This suggests that the PHBA_250_ chains are significantly more hydrophobic than the PHBA_150_ chains, which is consistent with the variable temperature ^1^H NMR spectroscopy studies (Figure 7).

**Figure 9:**
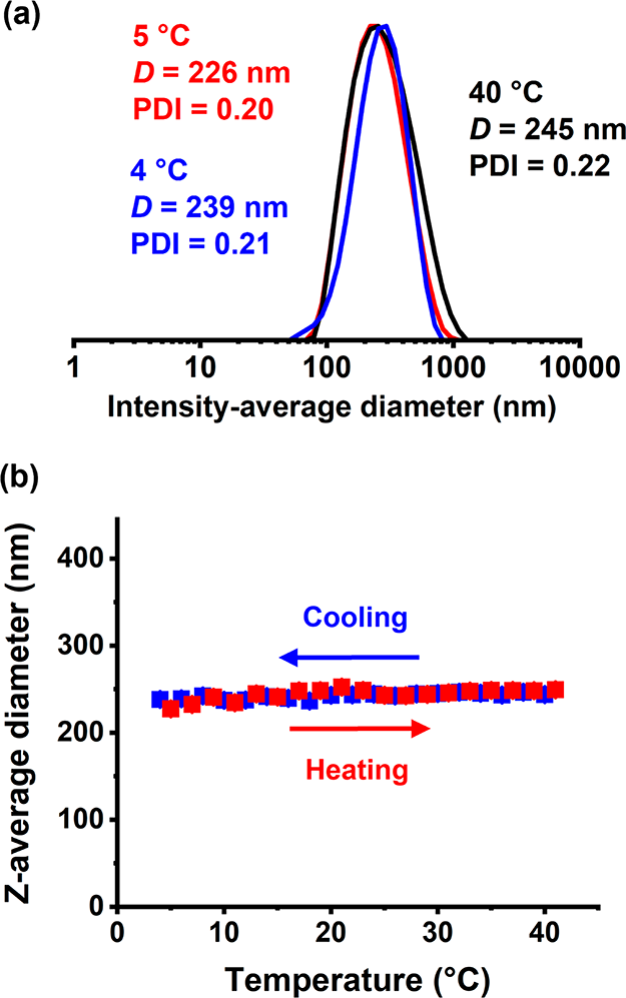
(a) Representative intensity-average particle size distributions observed at pH 6 for a 0.1% w/w aqueous dispersion of PHBA_250_ latex (prepared at 40% w/w solids using DDMAT). The red trace was recorded at 5 °C, the black trace after warming to 40 °C, and the blue trace on returning to 4 °C. (b) Variation in *z*-average diameter with temperature obtained for the same PHBA_250_ latex.

Remarkably, variable temperature DLS studies (Figure 9) conducted on a 0.1% w/w aqueous dispersion of PHBA_250_ latex particles at pH 6 indicated only rather weak thermoresponsive behavior. On heating from 5 to 40 °C, the *z*-average diameter increased from 226 nm to just 245 nm, indicating minimal nanoparticle core swelling.

**Scheme 2:**
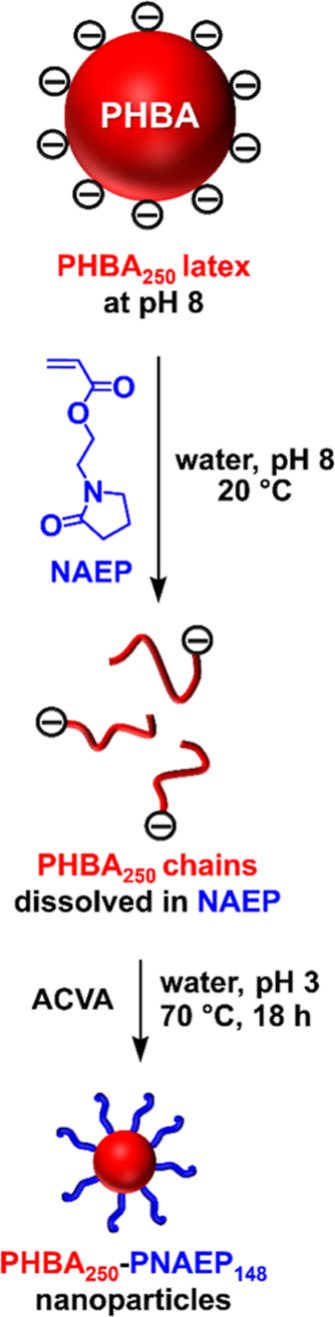
Schematic Representation Illustrating Reverse Sequence PISA Using an Anionic Charge-Stabilized PHBA_250_ Latex and NAEP Addition of this monomer causes in situ molecular dissolution of the latex particles while subsequent RAFT polymerization of NAEP leads to the formation of sterically stabilized PHBA–PNAEP nanoparticles at 54% w/w solids.

Given our recent success in using PHPMA latex particles to develop a new reverse sequence PISA formulation, we considered whether these PHBA latex particles could be employed in the same context. In principle, the synthesis of PHBA latex particles at 40% w/w solids should minimize the volume of the hydrophilic monomer [e.g., 2-(*N*-(acryloyloxy)ethyl pyrrolidone), NAEP; see Scheme 2] required for their molecular dissolution.^64^ Subsequent addition of a water-soluble initiator would then enable RAFT polymerization of this monomer from one end of the trithiocarbonate-capped PHBA chains. Initially, this would be a solution polymerization but as the NAEP is consumed, its ability to solubilize the weakly hydrophobic chains is progressively reduced. At some critical PNAEP DP (*x*), nucleation should occur to afford nascent sterically-stabilized PHBA_250_–PNAEP_*x*_ nanoparticles. Unlike conventional PISA, there should be no rate acceleration even if the cores of such nanoparticles become NAEP-swollen. Based on the data presented above, if such polymerizations were conducted to be at neutral pH, the terminal anionic carboxylate groups are likely to disrupt the desired self-assembly of the weakly hydrophobic PHBA_250_ chains. Hence the initial RAFT solution polymerization is best conducted at low pH, so the carboxylic acid group at the end of each PHBA_250_ chain remains in its neutral form (Scheme 2).

**Figure 10:**
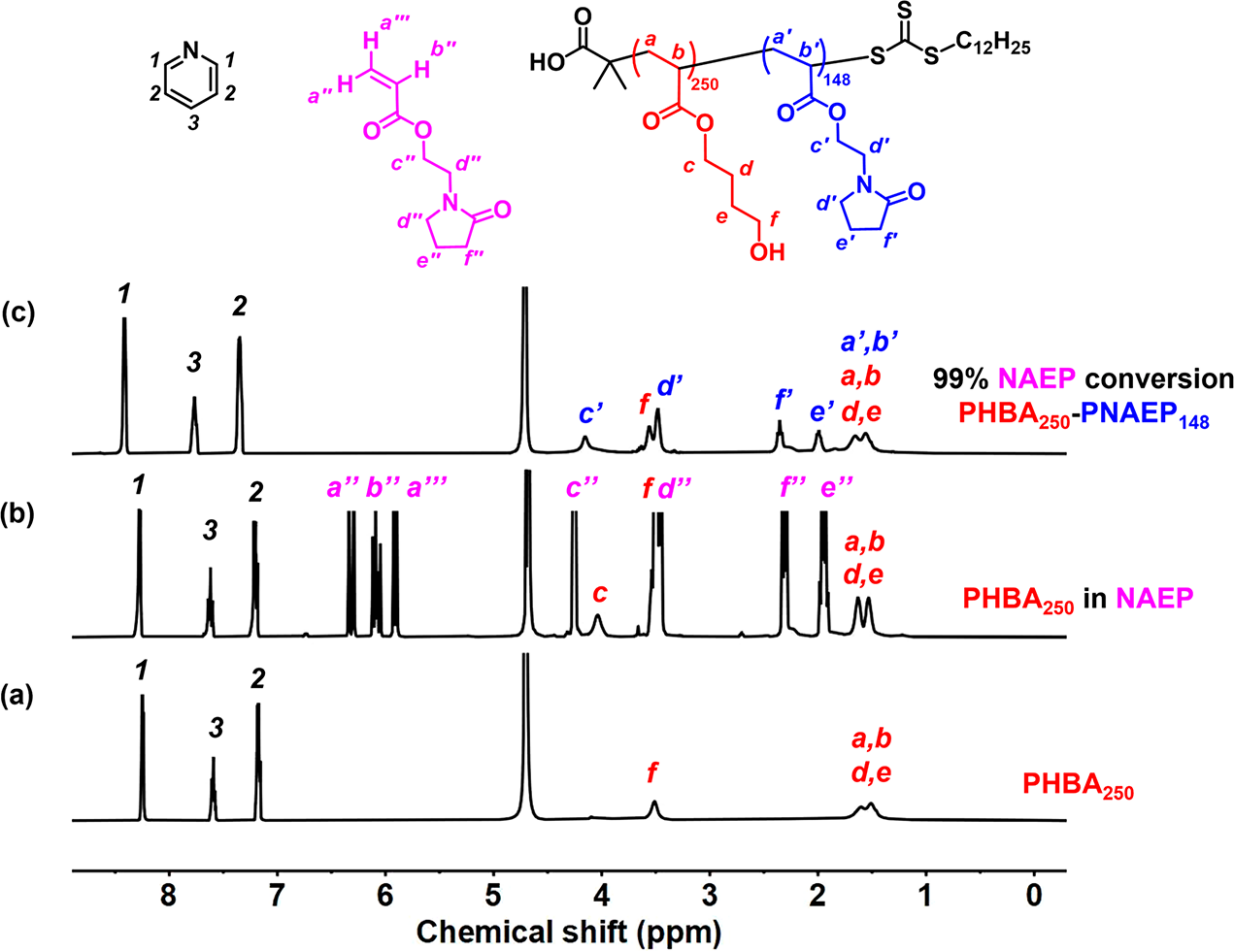
Assigned ^1^H NMR spectra recorded for (a) PHBA_250_ latex at pH 6 (D_2_O), (b) molecularly dissolved PHBA_250_ chains obtained at pH 3 (DCl/D_2_O) after addition of NAEP, and (c) same reaction mixture after NAEP polymerization, which affords PHBA_250_–PNAEP_148_ nanoparticles. All spectra were normalized using an external standard (pyridine).

**Figure 11:**
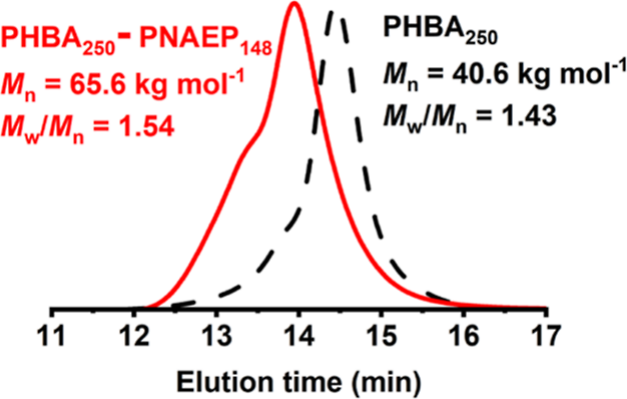
DMF GPC curves recorded for the PHBA_250_ precursor and the final PHBA_250_–PNAEP_148_ diblock copolymer chains.

As expected, addition of sufficient NAEP monomer to a 40% w/w aqueous dispersion of the PHBA_250_ latex causes its immediate molecular dissolution to form PHBA_250_ chains, as indicated by ^1^H NMR studies in D_2_O (Figure 10). The RAFT polymerization of NAEP was then conducted at pH 3 using ACVA initiator targeting a PNAEP DP of 148. This corresponds to the minimum amount of NAEP required to ensure molecular dissolution of the precursor PHBA_250_ (or PHBA_150_) latex particles. It is perhaps worth emphasizing that targeting such relatively long steric stabilizer chains inevitably means that only kinetically-trapped spheres can be targeted for this reverse sequence PISA formulation. ^1^H NMR spectroscopy studies of the final aqueous copolymer dispersion after 18 h at 70 °C indicated a final NAEP conversion of more than 99%. DMF GPC analysis indicated reasonably efficient chain extension of the PHBA_250_ precursor to produce PHBA_250_–PNAEP_148_ diblock copolymer chains (Figure 11).

**Figure 12:**
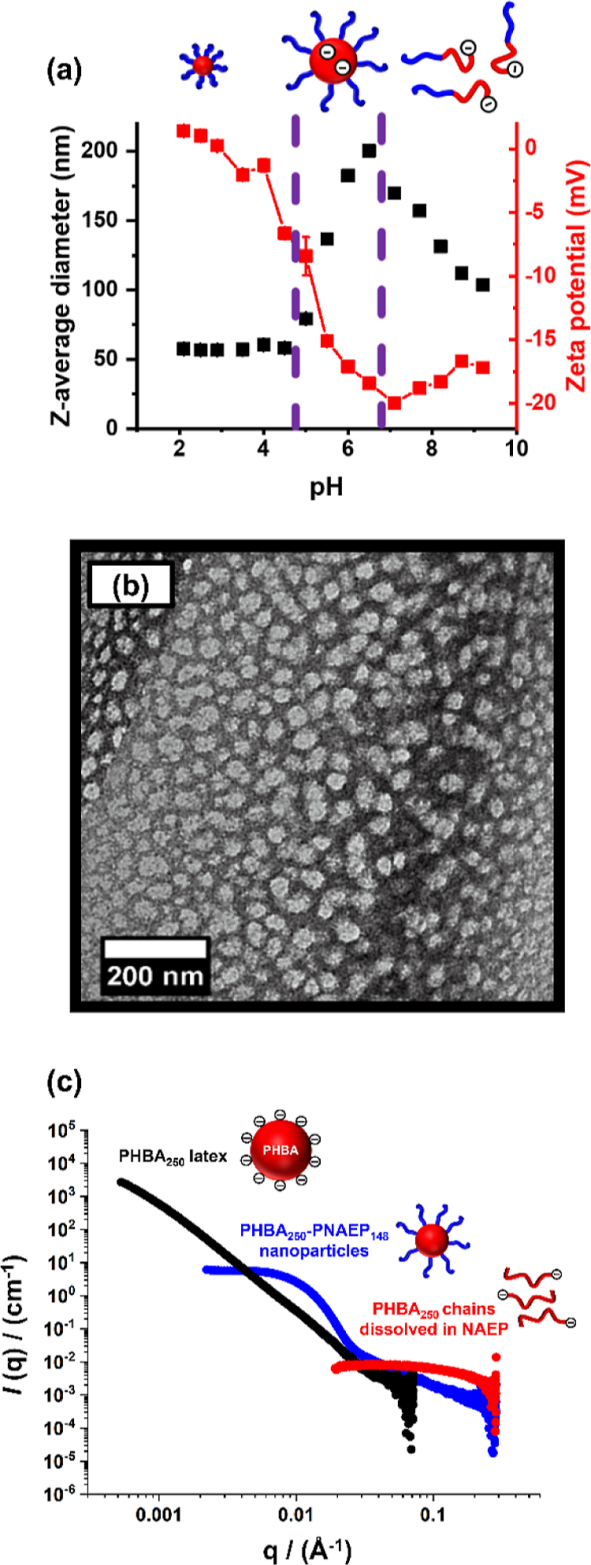
(a) Summary of DLS and zeta potential vs pH data for the PHBA_250_–PNAEP_148_ diblock copolymer nanoparticles. (b) TEM image of the final PHBA_250_–PNAEP_148_ diblock copolymer nanoparticles (after GA crosslinking of the PHBA_250_ cores). (c) SAXS patterns recorded for (i) a 5% w/w aqueous dispersion of the initial charge-stabilized anionic PHBA_250_ latex at pH 6 (black curve), (ii) the molecularly-dissolved PHBA_250_ chains obtained after addition of the NAEP monomer at pH 6 (red curve), and (iii) the final sterically-stabilized PHBA_250_–PNAEP_148_ nanoparticles at pH 3 (blue curve).

DLS was used to determine the pH dependence of the apparent *z*-average diameter for a 1.0% w/w aqueous dispersion of PHBA_250_–PNAEP_148_ nanoparticles (Figure 12a). PHBA_250_–PNAEP_148_ nanoparticles of 57 nm diameter were obtained between pH 2.0 and 4.4. Moreover, DLS studies confirmed that such nanoparticles remained colloidally stable at pH 3 in the presence of 0.1 M MgSO_4_ or after a freeze–thaw cycle conducted in the absence of added salt (Table S1). These observations suggest that the highly hydrophilic PNAEP_148_ chains confer steric stabilization, whereas the weakly hydrophobic PHBA_250_ chains are located within the nanoparticle cores. A TEM image of such nanoparticles after core-crosslinking using glutaraldehyde is shown in Figure 12b. Above pH 4.4, the terminal carboxylic acid groups on the PHBA_250_ chains located within the nanoparticle cores become deprotonated, as indicated by the more negative zeta potential. ^1^H NMR studies (Figure S6) indicate partial swelling and hydration of the PHBA cores under such conditions, which leads to an increase in apparent *z*-average diameter from 57 nm up to 200 nm (Figure S7). Above pH 6.5, the nanoparticles dissociate to form molecularly-dissolved PHBA chains owing to the build-up of anionic charge within their cores. The apparent increase in *z*-average diameter observed above pH 6.5 is attributed to weak inter-chain interactions. There is no doubt that molecular dissolution occurs above pH 6.5 because a substantial reduction in the scattered light intensity (or derived count rate) is observed (see Figure S8). This is consistent with the ^1^H NMR spectra shown in Figure S6. Finally, DLS studies confirmed that the sterically-stabilized PHBA_250_–PNAEP_148_ diblock copolymer nanoparticles did not exhibit any discernible thermoresponsive behavior (Figure S9).

A reverse sequence PISA synthesis was also attempted with the PHBA_150_ latex at pH 3 using the minimum amount of NAEP monomer required to dissolve these latex particles (which corresponded to a target PNAEP DP of 79). Again, ^1^H NMR spectroscopy studies confirmed essentially full NAEP conversion, while DMF GPC studies indicated a relatively high blocking efficiency for the final PHBA_150_–PNAEP_79_ nanoparticles (Figure S10a). However, DLS analysis indicated a bimodal particle size distribution in this case, which suggests a mixture of nanoparticles and molecularly-dissolved copolymer chains (Figure S10b).

Small-angle X-ray scattering patterns were recorded for (i) a 5% w/w aqueous dispersion of the initial charge-stabilized anionic PHBA_250_ latex at pH 6 (black curve), (ii) the molecularly-dissolved PHBA_250_ chains formed after the addition of the NAEP monomer (red curve), and (iii) the final sterically-stabilized PHBA_250_–PNAEP_148_ nanoparticles at pH 3 (blue curve). These three patterns are shown on an absolute intensity scale in Figure 12c. Hence the five orders of magnitude reduction (from approximately 3000 cm^–1^ to around 10^–2^ cm^–1^) in scattering intensity, *I*(*q*), that occurs on addition of the NAEP monomer is consistent with the formation of molecularly-dissolved PHBA_250_ chains. Moreover, subsequent chain extension of these precursor chains with NAEP clearly leads to the formation of relatively small spherical nanoparticles at pH 3, as judged by the zero gradient observed in the low *q* region and the near thousand-fold increase in *I*(*q*) from approximately 10^–2^ cm^–1^ up to around 10 cm^–1^. The mean PHBA-core diameter for the PHBA_250_–PNAEP_148_ nanoparticles at pH 3 is estimated to be around 30 nm using the well-known relation *d* = 4.49/*q*_min_, where *q*_min_ is approximately 0.03 Å^–1^ and *d* is a real-space distance corresponding to the particle radius (see blue curve). This value is reasonably consistent with the hydrodynamic *z*-average diameter of 57 nm reported by DLS for these nanoparticles. Inspecting the TEM image shown in Figure 12b, the glutaraldehyde-stabilized nanoparticles are most likely only lightly crosslinked and hence prone to a certain degree of deformation or flattening during sample grid preparation.

## Conclusions

RAFT aqueous dispersion polymerization of HBA using a carboxylic acid-functionalized RAFT agent at pH 8 leads to the efficient formation of anionic PHBA latex particles. The soft film-forming nature of the low-*T*_g_ PHBA chains means that covalent stabilization is required prior to TEM analysis: this imaging technique reveals a polydisperse spherical morphology. Such charge-stabilized latexes comprise low-dispersity PHBA chains and exhibit dual stimulus-responsive behavior. For example, variable temperature ^1^H NMR studies indicate that such latexes become highly swollen on heating owing to partial solvation of the weakly hydrophobic HBA repeat units. Moreover, latex aggregation occurs on lowering the solution pH owing to the loss of surface charge as the anionic carboxylate groups become protonated. On the other hand, latex dissolution occurs in alkaline media. Such PHBA latexes can also be molecularly dissolved on addition of a suitable water-soluble monomer such as NAEP. This enables the development of a new reverse sequence PISA formulation, which produces relatively small sterically stabilized PHBA–PNAEP nanoparticles. This approach is complementary to the two other reverse sequence PISA synthesis routes recently reported by our group.

## Supporting Information Available

Additional DLS data obtained under various conditions; digital photograph recorded for PHBA_150_ latex at pH 6 and pH 8; additional TEM images recorded before and after a thermal cycle; variable temperature ^1^H NMR spectra recorded for PHBA_250_ latex; ^1^H NMR spectra recorded for PHBA_250_-PNAEP_148_ nanoparticles; additional GPC data obtained for PHBA_150_ and PHBA_150_-PNAEP_79_ (PDF)
