# Supplementary material for: Synthesis and Characterization of Charge-Stabilized Poly(4-hydroxybutyl acrylate) Latex by RAFT Aqueous Dispersion Polymerization: A New Precursor for Reverse Sequence Polymerization-Induced Self-Assembly
Source: Macromolecules. 2023 Jun 2;56(11):4296–306. doi: 10.1021/acs.macromol.3c00534 (PMC10273316; doi:10.1021/acs.macromol.3c00534)
Supplement: Supplementary file 1 — ma3c00534_si_001.pdf [file ma3c00534_si_001.pdf]

Supporting Information for

Synthesis and Characterization of Charge-Stabilized Poly(4-Hydroxybutyl Acrylate) Latex by RAFT Aqueous Dispersion Polymerization: A New Precursor for Reverse Sequence Polymerization-Induced Self-Assembly

Hubert Buksa[a], Thomas J. Neal[a], Spyridon Varlas[a],

Saul J. Hunter[a], Osama M. Musa[b] and Steven P. Armes[a,*]

a. Department of Chemistry, University of Sheffield,

Brook Hill, Sheffield, South Yorkshire, S3 7HF, UK.

b. Ashland Specialty Ingredients, 1005 US 202/206,

Bridgewater, New Jersey, 08807, USA.

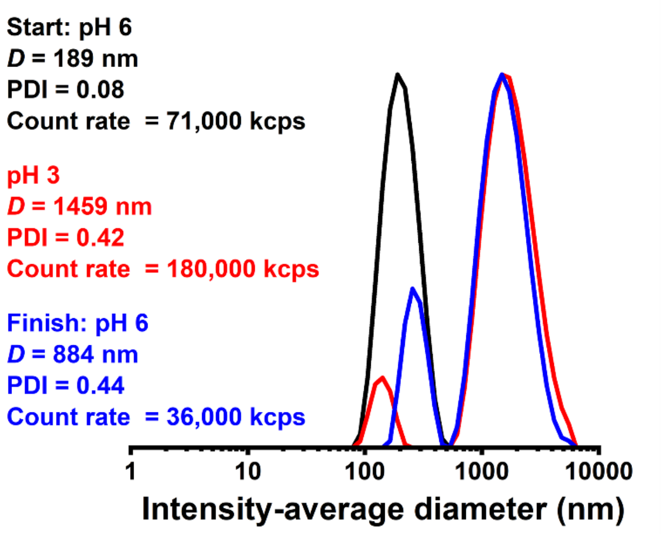

Figure S1. DLS studies of the pH-responsive behavior of a 0.1% w/w aqueous dispersion of a $PHBA_{150}$ latex during a pH 6 to pH 3 to pH 6 cycle. Irreversible latex aggregation occurs at pH 3.

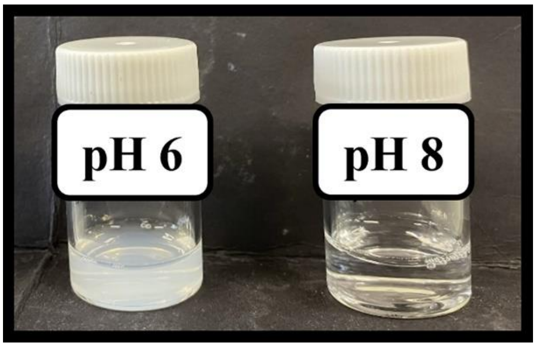

Figure S2. Digital photograph recorded for a 0.1% w/w aqueous dispersion of an anionic $PHBA_{150}$ latex (left) at pH 6 and (right) at pH 8.

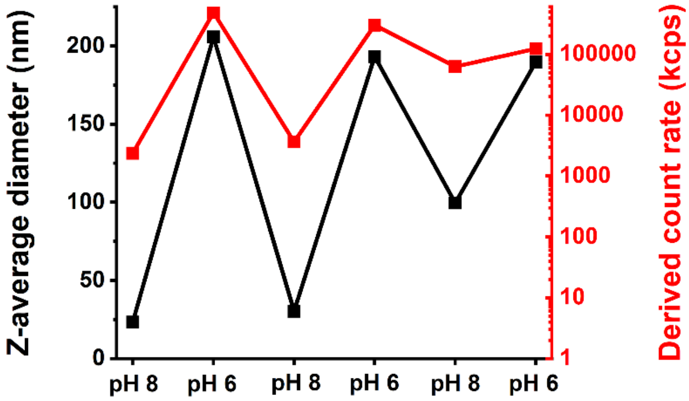

Figure S3. Variation in z-average diameter for $PHBA_{150}$ latex/chains observed during two pH cycles according to DLS studies performed at 0.1% w/w solids.

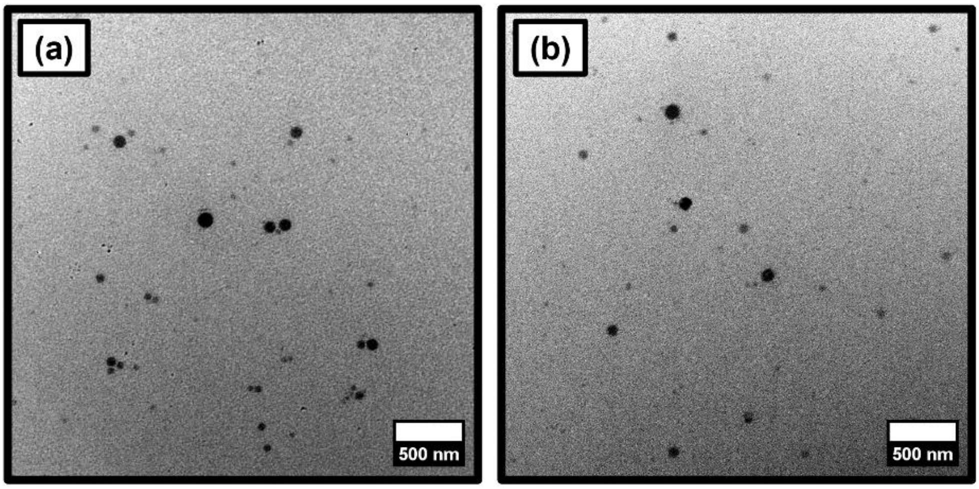

Figure S4. TEM images recorded for a 0.1% w/w aqueous dispersion of $PHBA_{150}$ latex particles at pH 6 after glutaraldehyde crosslinking at 5 °C before (a) and after (b) a 5 °C to 41 °C to 4 °C thermal cycle.

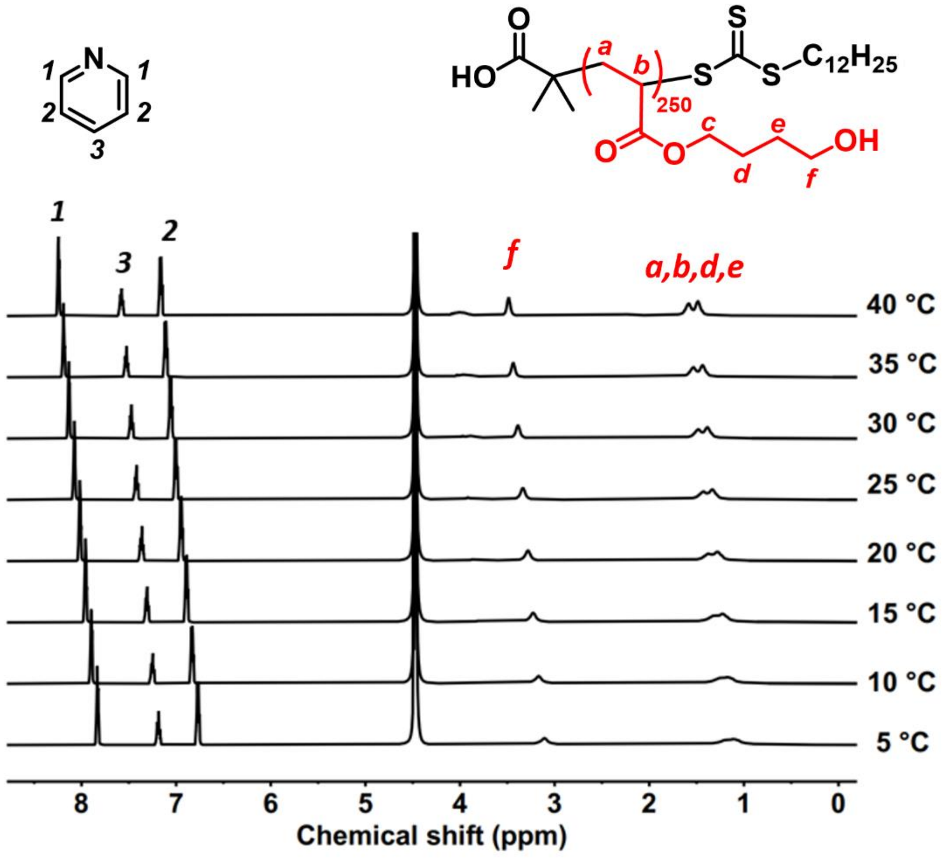

Figure S5. (a) Variable temperature ^{1}H NMR spectra recorded from 5 °C to 40 °C for a 5.0% w/w aqueous dispersion of $PHBA_{250}$ latex particles in D_2O at pH 6. All spectra were normalized using an external standard (pyridine).

Table S1. DLS data recorded for a 1.0% w/w aqueous dispersion of $PHBA_{250}$-$PNAEP_{148}$ nanoparticles at pH 3, after one freeze-thaw cycle for this acidic dispersion, and in the presence of 0.1 M $MgSO_4$ at pH 3. These observations are consistent with a steric stabilization mechanism for these nanoparticles.

Dispersion conditions	DLS diameter (nm)	DLS polydispersity
pH 3	60	0.11
After freeze-thaw at pH 3	60	0.12
0.1 M $MgSO_4$ at pH 3	71	0.10

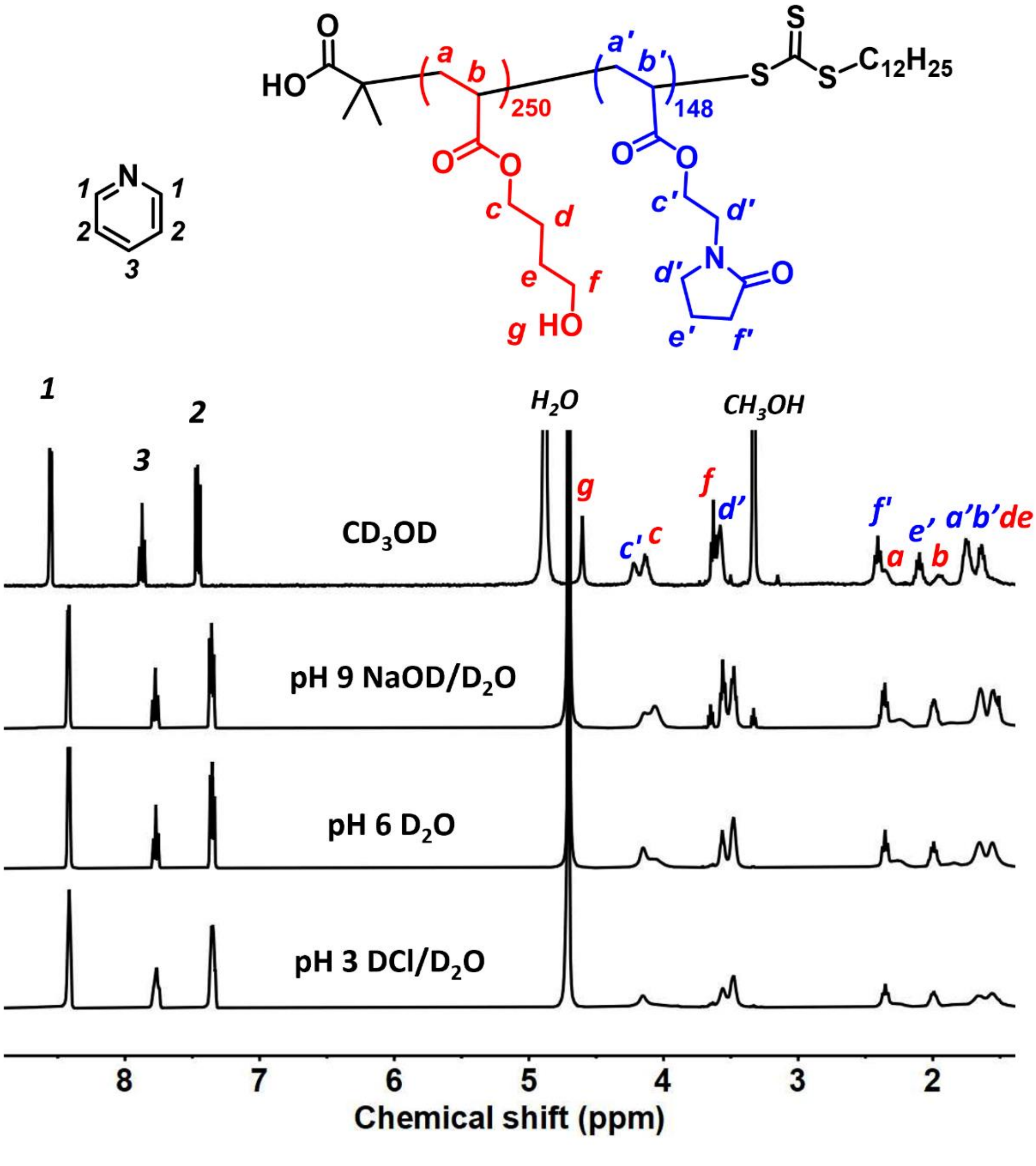

Figure S6. ^{1}H NMR spectra recorded for a 5.0% w/w aqueous dispersion of PHBA$_{250}$-PNAEP$_{148}$ nanoparticles particles at pH 3 (DCl/D_2O), pH 6 (D_2O) and pH 9 (NaOD/D_2O) and for the molecularly-dissolved PHBA$_{250}$-PNAEP$_{148}$ diblock copolymer in CD_3OD. All spectra were normalized using an external standard (pyridine).

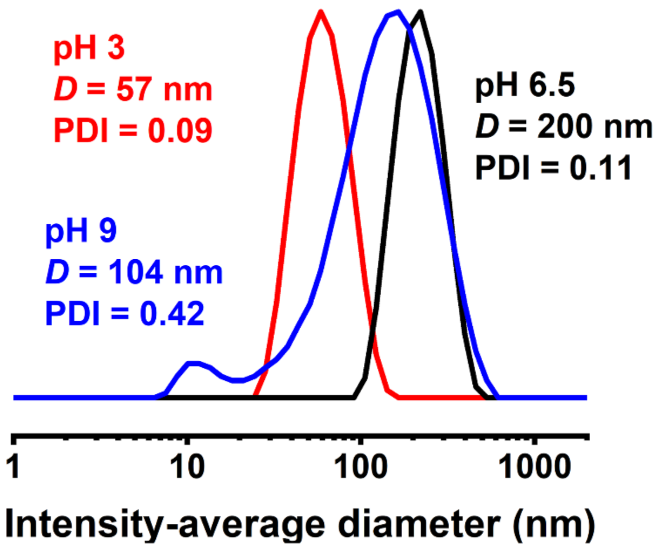

Figure S7. DLS particle size distributions recorded for a 1.0% w/w aqueous dispersion of $PHBA_{250}$-$PNAEP_{148}$ nanoparticles at pH 3, pH 6.5 and pH 9.

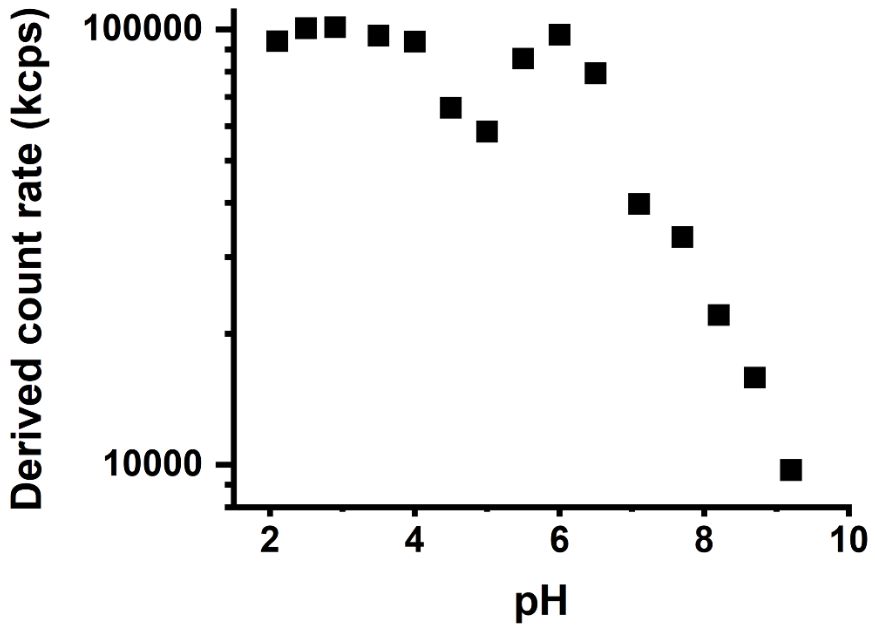

Figure S8. pH dependence of the scattered light intensity (derived count rate) determined during DLS studies of a 0.1 % w/w aqueous dispersion of $PHBA_{250}$-$PNAEP_{148}$ nanoparticles. The substantial reduction in derived count rate observed above pH 6.5 indicates that molecular dissolution occurs under such conditions.

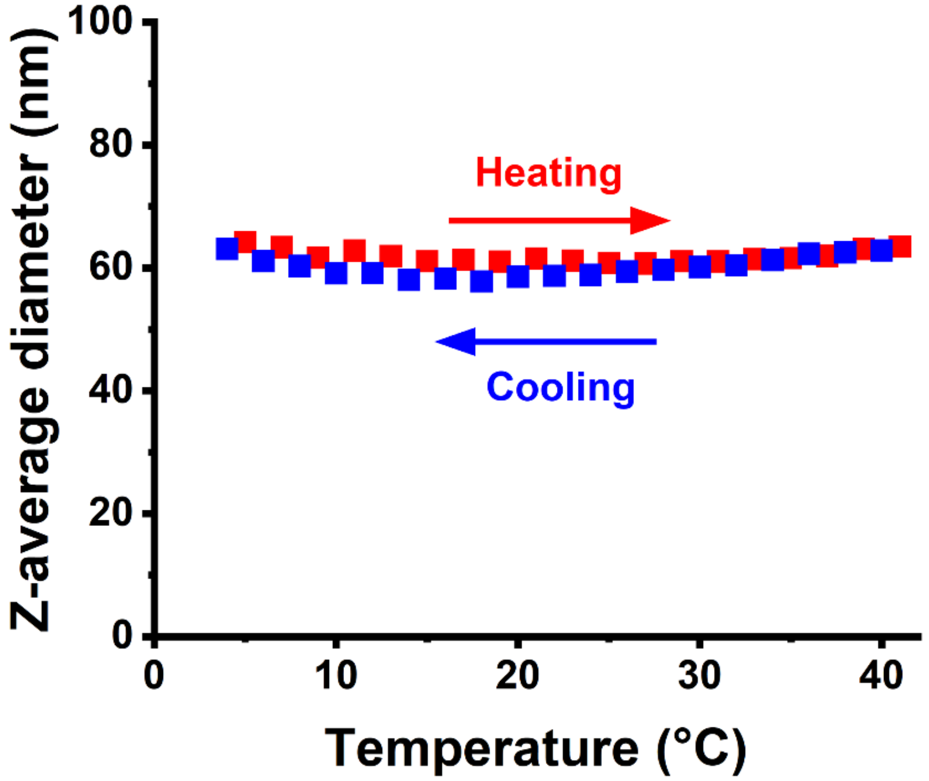

Figure S9. Variation in z-average diameter with temperature for a 0.1% w/w aqueous dispersion of $PHBA_{250}$-$PNAEP_{148}$ nanoparticles recorded at pH 3.

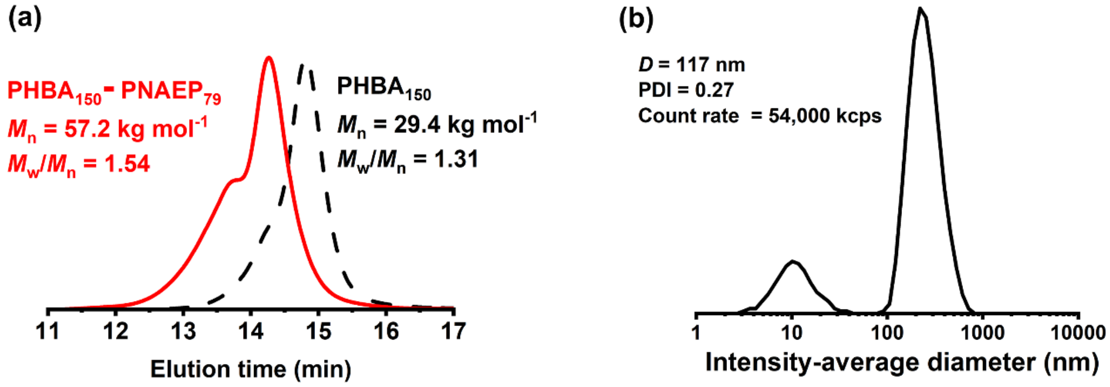

Figure S10. (a) DMF GPC curves recorded for the $PHBA_{150}$ precursor and the final $PHBA_{150}$-$PNAEP_{79}$ diblock copolymer after reverse sequence PISA performed at pH 3. (b) DLS data obtained for a 0.1% w/w aqueous dispersion of these $PHBA_{150}$-$PNAEP_{79}$ nanoparticles at pH 3.
